# Supplementary material for: Neuronal evidence for good-based economic decisions under variable action costs
Source: Nat Commun. 2019 Jan 23;10:393. doi: 10.1038/s41467-018-08209-3 (PMC6344483; doi:10.1038/s41467-018-08209-3)
Supplement: Supplementary file 1 — Supplementary Information [file 41467_2018_8209_MOESM1_ESM.pdf]

Neuronal Evidence for Good-Based Economic Decisions
Under Variable Action Costs

Xinying Cai and Camillo Padoa-Schioppa

Supplementary Information

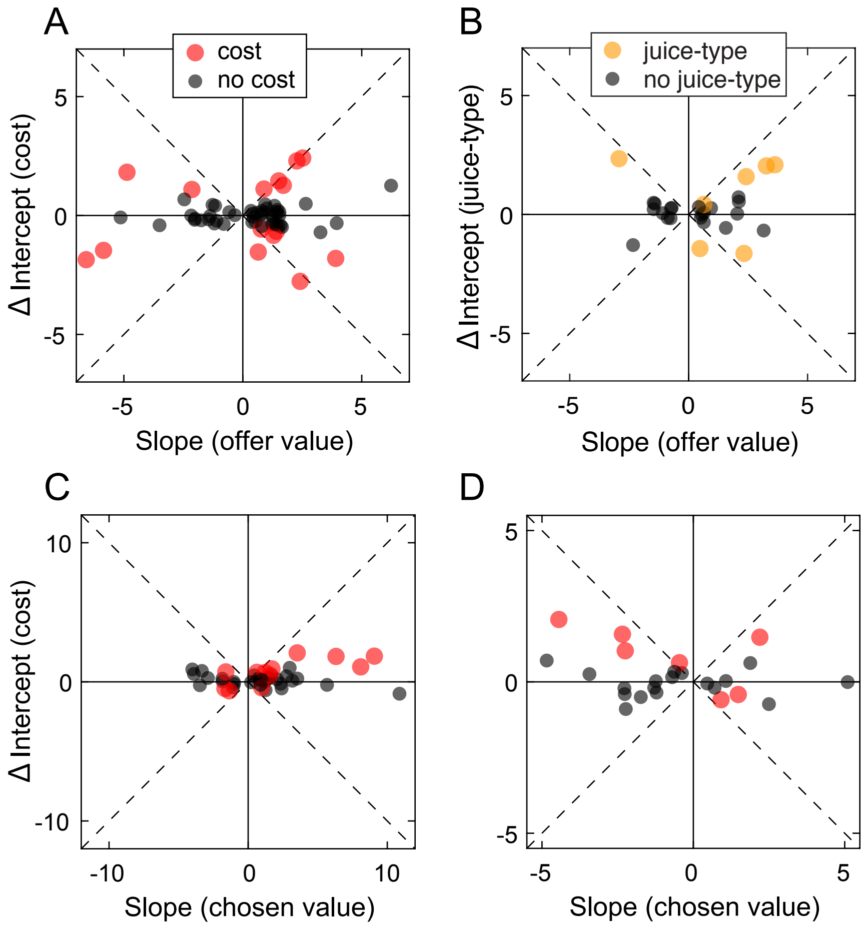

Supplementary Figure 1. Analysis of covariance. (A) Offer value (juice) responses in post-offer time window. The x-axis represents the effect of offer value (slope) and the y-axis represents the effect of cost (difference in intercept). The effect of cost was statistically significant ($p<0.05$) for 15/60 (25%) responses. This effect could be congruent (8 responses) or incongruent (7 responses) with the effect of offer value (juice). Across the population, there was no significant correlation between the slope of the encoding and the cost-related offset (Pearson correlation, $r=0.12$, $p=0.36$). (B) Offer value (cost) responses in post-offer time window. The x-axis represents the effect of offer value (slope) and the y-axis represents the effect of juice type (difference in intercept) other than that contributed through the indifference point (ρ). This effect was statistically significant ($p<0.05$) for 7/27 (26%) responses. Thus effect could be congruent (4 responses) or incongruent (3 responses) with the effect of offer value (juice). Across the population, there was no significant correlation between the slope of the encoding and the juice type-related offset (Pearson correlation, $r=0.11$, $p=0.57$). (C) Chosen value responses, post-offer time window. In this time window, the effect of cost was statistically significant for 12/45 (27%) responses. This effect could be congruent (2 responses) or incongruent (10 responses) with the effect of chosen value. Across the population, there was no significant correlation between the slope of the encoding and the cost-related offset (Pearson correlation, $r=0.21$, $p=0.16$). (D) Chosen value responses, post-target time window. In this time window, the effect of cost was statistically significant for 7/25 (28%) responses. In this case, however, the effect was mostly congruent with the effect of chosen value (6/7 responses). The slope of the encoding and the cost-related offset were not correlated across the population (Pearson correlation, $r=-0.27$, $p=0.19$).
